# An Investigation of *TDA1* Deficiency in *Saccharomyces cerevisiae* During Diauxic Growth

**DOI:** 10.1002/yea.4004

**Published:** 2025-06-26

**Authors:** Erik Y. Bjurström, Praphapan Lasin, Daniel Brunnsåker, Ievgeniia A. Tiukova, Ross D. King

**Affiliations:** ^1^ Department of Life Sciences Chalmers University of Technology Göteborg Västra Götaland Sweden; ^2^ Department of Computer Science and Engineering Chalmers University of Technology Göteborg Västra Götaland Sweden; ^3^ Division of Industrial Biotechnology KTH Royal University of Technology Stockholm Stockholm Sweden; ^4^ Department of Chemical Engineering and Biotechnology University of Cambridge Cambridge Cambridgeshire UK; ^5^ Alan Turing Institute London Greater London UK

**Keywords:** diauxic growth, differential gene expression, glucose repression, mitochondrial translation, TDA1, transcriptomics

## Abstract

Tda1p is a protein kinase in *Saccharomyces cerevisiae*. Here we investigate the function of *TDA1* during the diauxic shift using transcriptomics. We compared the gene expression in the deletion mutant *tda1∆* and the reference strain (BY4741) during both the aerobic fermentation phase (log phase), and the respiratory phase (post‐diauxic shift phase, PDS) in three separate independent experiments. We found: Differential gene expression analysis showed that compared to the reference strain, the *tda1∆* mutant exhibited an upregulation of the glucose repressed hexose transporter *HXT6* during the log phase, and upregulation of mitochondrial proteins and genes related to mitochondrial translation during the PDS phase. Gene set enrichment analysis showed an enrichment in mitochondrial translation in the PDS phase for the deletion mutant *tda1∆*, but not for the reference strain. Transcription factor analysis showed that the enrichment of Mig1p repressed genes was not statistically significant in *TDA1* deletion mutants for neither log‐phase nor PDS‐phase. This conflicted with the previously suggested model that argued for an interaction between Tda1p and Mig1p. Instead, transcription factor analysis showed an enrichment of genes regulated by the HAP‐complex, which regulates mitochondrial translation, during the PDS‐phase in the tda1∆ mutant. The combined evidence from this study indicates that Tda1p does not participate in Mig1p‐mediated glucose repression. Instead, we propose that it is involved in the regulation of mitochondrial translation by repressing the expression of HAP complex subunits.

## Summary

- This study investigated *TDA1*, a kinase with disputed biological function, using transcriptomics data.
- Genes related to “Mitochondrial translation” were enriched in *tda1∆* post‐diauxic shift.
- A subunit of the HAP‐complex, which induces mitochondrial translation, was upregulated in *tda1∆*.
- Genes targeted by the HAP‐complex were enriched in *tda1∆*.
- The combined evidence suggests that *TDA1* regulates mitochondrial translation during the diauxic shift by repressing the HAP‐complex.

## Graphical abstract

Tda1p is a protein kinase in *Saccharomyces cerevisiae* with unknown/disputed function. Its role during the diauxic shift was investigated in three independent transcriptomics experiment. From the evidence that was consistent across the experiments, we propose that *TDA1* is involved in regulation of mitochondrial translation by repressing the expression of HAP‐complex subunits.

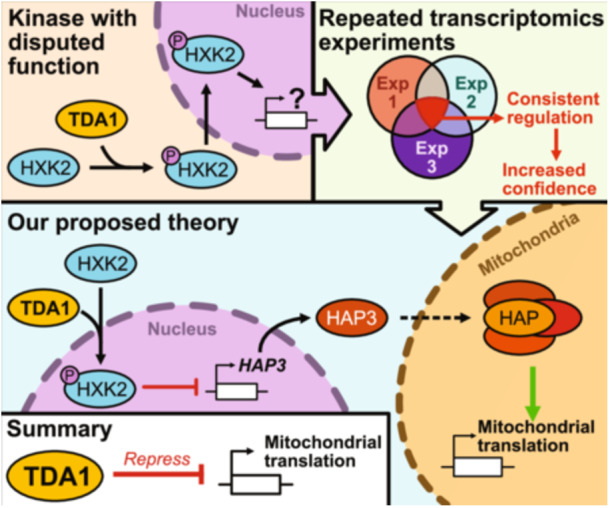

## 1 Introduction

*Saccharomyces cerevisiae* is the best‐studied eukaryotic model organism for research on transcriptomic and metabolomic regulation during stress and metabolic adaptations. An important example of a metabolic adaptation of *S. cerevisiae* is the diauxic shift (De Deken 1966; Hagman et al. 2013). When glucose is available in high concentration, a *S. cerevisiae* population grows exponentially in a phase known as the logarithmic (log) phase that is characterised by production of ethanol through glycolysis and alcoholic fermentation (De Deken 1966). As the glucose level decreases, the cells enter the diauxic shift, in which the cells enter a quiescent state and begin consumption of nonfermentable carbon sources, that is, ethanol (Miles et al. 2021). During the post‐diauxic shift (PDS) phase, the metabolism shifts to a respiratory pathway that includes the glyoxylate shunt and tricarboxylic acid cycle (Schlossarek et al. 2022).

The expression of respiratory genes is regulated in *S. cerevisiae* through glucose repression (Kayikci and Nielsen 2015; Ronne 1995). When glucose is abundant, genes responsible for catabolism of nonfermentable carbon sources are repressed by the transcription repressor Mig1p in the nucleus (Kayikci and Nielsen 2015; Ronne 1995). Conversely, when glucose levels are exhausted, Mig1p is dephosphorylated and deactivated through the Snf1p signalling network, consequently relieving the cell of glucose repression (Vallier and Carlson 1994). Another presumed regulatory mechanism that occurs during the diauxic shift is the phosphorylation of Hxk2p (Kaps et al. 2015; Müller et al. 2022). Hx2p is the predominant hexose kinase involved in the early stages of glycolysis and is therefore a crucial enzyme for fermentative growth on glucose (Rodríguez et al. 2001). Hxk2p exists in a homodimer‐monomer equilibrium, where the equilibrium is forced towards the monomer form upon phosphorylation at the serine 15 site by the protein kinase Tda1p (Lesko et al. 2023).

It has been argued in some studies that the phosphorylated Hxk2p also acts as a transcription factor during glucose repression. This theory hypothesises that Hxk2p localises into the nucleus and interacts with the Mig1p complex during high glucose levels, and is excluded from the nucleus as glucose levels decrease (Kaps et al. 2015). In other studies it is argued that Hx2kp does not associate with Mig1p (Lesko et al. 2023). Instead, Hxk2p localises in the cytosol during high glucose concentrations, and is then imported into the nucleus as glucose depletes (Lesko et al. 2023). This behaviour is similar to the one found in other mammalian cells (Lesko et al. 2023). Recent evidence from confocal microscopy studies of GFP‐tagged Hxk2p suggests that the latter model is more accurate, as it has been shown that Hxk2p is indeed localised in the cytosol during the log‐phase and is found more abundantly in the nucleus during the PDS‐phase (Lesko et al. 2023). Furthermore, gene deletion studies have shown that the abundance of unphosphorylated Hxk1p and Hxk2p were significantly lower in *TDA1* deletion strains, yet no significant decrease in cell fitness for these deletion strains during low glucose levels could be observed (Müller et al. 2022). This observation challenges the theory that Tda1p is essential in the regulatory pathway for respiratory genes since the Mig1p mediated model postulates that strains lacking *TDA1* would suffer from impaired growth during glucose depletion (Kaps et al. 2015). Therefore, the current consensus is that the molecular function of Tdad1p is to phosphorylate Hxk2p during glucose starvation, upon which Hxk2p is localised into the nucleus. However, the biological process of Tda1p and the function of Hxk2p nuclear localisation remain unknown, the former model suggests Mig1p involvement while the latter rejects Mig1p involvement without providing a biological function.

There is therefore a need for studies generating and analysing omics datasets obtained in vivo, to understand the gene expression profile and regulation specific to Tda1p to elucidate its biological role. Previously, a metabolomics study investigating diauxic growth of *S. cerevisiae*, which included *TDA1* deletion strains, was performed (Brunnsåker et al. 2023). The key findings for the *tda1∆* strain was enriched activity in the purine metabolic pathways during the log‐phase and the sphingolipid biosynthesis pathway during the PDS‐phase.

This study aims to investigate the biological role of *TDA1* using transcriptomic analysis.

Differential expression analysis was performed to investigate the biological process of *TDA1* during the diauxic shift. Studying transformative metabolic adaptations, such as the diauxic shift, necessitates working in dynamical systems (i.e. batch growth) since it is difficult, if not impossible, to emulate diauxic shifts in stable systems such as chemostats (Brauer et al. 2005). Therefore, to address reproducibility concerns that arise from cultivation conditions, the experiment was performed three times to assess the reproducibility of the experiments and ensure that any conclusions drawn from the results were robust.

## 2 Methods and Materials

Differential expression analysis was performed to investigate the biological process of *TDA1* during the diauxic shift. To demonstrate the reproducibility and robustness of the results we performed the experiment three separate times.

### 2.1 Strain Selection and Cultivation Conditions

The *S. cerevisiae* reference BY4741 (Accession number: Y00000) and single‐gene deletion strain BY4741 *tda1∆/YMR291W* (Y00878) were taken from the yeast deletant library, provided by EUROSCARF (Giaever and Nislow 2014). The strains were revived from −80°C glycerol stocks by overnight‐cultivation in YPD media at 30°C, 220 rpm. The strains were then streaked out on YPD plates and incubated at 30°C for 3 days. Single colonies were then used to inoculate pre‐cultures containing YPD (2% (w/v) dextrose) for 15 h at 30°C, 220 rpm. Finally, the main cultivations were performed in 250 mL wide‐necked baffled shake flasks, with a working volume of 40 mL SC medium (6.7 g/L YNB without amino acids and with ammonium sulphate, 1x Amino acid mix, 2% (w/v) dextrose). Each culture was inoculated with an initial OD600 of 0.05, and subsequently incubated at 30°C, 220 rpm. The *tda1∆* and reference strain of *S. cerevisiae* were grown in biological triplicates (first experiment) or with six replicates (second and third experiment) under the same conditions using wide‐necked baffled shake flasks.

**Figure 1:**
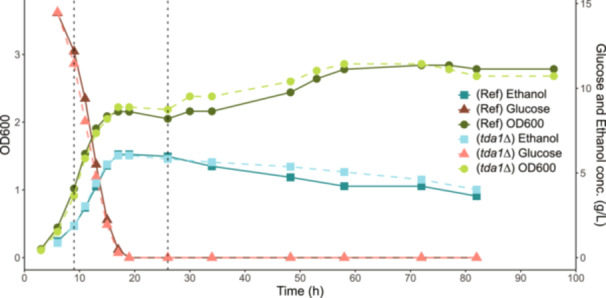
Growth curves and extracellular media over time of the *tda1∆* mutant and reference strain. The green lines represent OD600, an indirect value of biomass accumulation, over time. The red lines represent glucose concentration over time while the blue lines represent ethanol concentration over time. The solid lines are measurements of the reference strain while the dashed lines belong to the *tda1∆* mutant. The vertical dotted line represents the time when sampling took place (log phase sample = 9 h post inoculation, PDS phase sample = 26 h post inoculation). The cultivations were performed with three biological replicates (*n* = 3). Each point in the graph is the result of a rolling median (*k* = 4) of the median value from each time point.

The cultures were sampled for OD600, RNAseq, and HPLC after 9 h (pre‐diauxic shift) and 26 h (post‐diauxic shift) postinoculation, see Supplementary Table 1. Additionally, a growth curve was obtained by replicating the above experiment using the same conditions, see Figure 1.

### 2.2 RNAseq Extraction and Analysis

1.0–1.5 mL cell broth with an adjusted OD600 of 10 was harvested using centrifugation (5,000 rcf, 5 min) and RNA was then immediately extracted using an RNeasy™ kit (QIAGEN 74104). The extracted RNA was stored in 30 μL RNAse free water at −80°C. Total RNA quantity was measured using NanoDrop™ and the quality was estimated using BioAnalyzer™. The library construction was performed using the Illumina TruSeq Stranded mRNA (polyA) kit. The samples were sequenced using NovaSeq. 6000 with an average read depth of 55.8 Mreads per sample for the first experiment and 34.13 Mreads per sample for experiment two and three. The library construction and sequencing for experiment one was performed by NGI in Solna, Sweden. The library construction and sequencing for experiment two and three were performed by Azenta in Leipzig, Germany. Independent sequencing helps ensure the reproducibility of the results. Data are deposited at European Nucleotide Archive (PRJEB59812). The raw. FASTQ‐files were processed using the nf‐core/rnaseq v3.10.1 pipeline (Harshil Patel et al. 2023), using Ensembl entry R64‐1‐1 (*S. cerevisiae*) as a reference genome, STAR (Dobin et al. 2013) for fragment aligning and Salmon (Patro et al. 2017) for quantification. Samples that did not meet the required RNA amount (100 ng) for the library preparation were discarded.

### 2.3 HPLC Analysis

Extracellular metabolites were analysed using HPLC. 700 μL cell broth was centrifuged (5,000 rcf, 5 min), after which the cell pellet was discarded. The supernatant was then stored at −80°C. Before HPLC analysis, the supernatant was centrifuged again (12,000 rcf, 10 min). HPLC was performed using ThermoFisher/Dionex UltiMate 3000 HPLC system with the Chromeleon 6.80 software for experiment one and Shimadzu Nexera 2040 with the LabSolutions CS software for experiment two and three. The extracellular metabolites were quantified using a serial dilution of a standard containing glucose and ethanol.

### 2.4 Differential Gene Expression Analysis

Raw RNA data was analysed using the DESeq. 2 software package (Love et al. 2014). Raw expression data (Supplementary Table 2.1) was normalised, fit to a negative binomial distribution, and the log2‐fold change of low expression genes was adjusted using the DESeq. 2‐package in R. Hypothesis testing was performed using the Wald test and were corrected for false positives using FDR/Benjamini‐Hochberg method with a cut‐off of adjusted *p*‐value < 0.05. The following contrasts were used for this study: *tda1∆* versus reference during log phase, *tda1∆* versus reference during PDS phase, PDS phase versus log phase for reference, and PDS phase versus log phase for *tda1∆*. The log2 fold changes (Log2FC) were shrunk using DESeq. 2's lfcShrink function with the ‘ashr’ setting (Stephens 2016). The differential gene expression for each experiment was performed separately.

### 2.5 Gene Set Enrichment Analyses

Gene‐set analysis of the differential expression caused by *TDA1* deletion during the diauxic shift was performed using the Fast Gene Set Enrichment Analysis (FGSEA) (Korotkevich et al. 2016), and the contrasts obtained from the differential expression analysis. The gene sets were defined by GO‐slim term annotations obtained from the *Saccharomyces cerevisiae* Genome Database (SGD), version 2024‐11‐11 (Cherry et al. 2012). The cut‐off for significant enrichment was set at an adjusted *p*‐value < 0.01.

Transcription factor enrichment analysis of Mig1p and Hap4p was performed using the contrasts from the differential expression analysis. The set of genes regulated the transcription factor were obtained by Yeastract+ (Teixeira et al. 2023), 2024‐11‐11. Only genes with both direct DNA binding evidence and expression evidence were included in the analysis. The consensus enrichment analysis was performed using the Fisher's exact test, the Boschloo's test, and the FGSEA (Boschloo 1970; Subramanian et al. 2005). These methods were used because previous studies has found that Fisher's exact test (and by extension Boschloo's test) tend to be conservative (excessive false negatives) while GSEA under some circumstance misidentifies gene sets as statistically significant when the observed expression is uncorrelated to a phenotype (excessive false positives) (Abatangelo et al. 2009; Dinu et al. 2007). Thus, the consensus results from these methods should provide a balanced interpretation of the biology being studied. The contingency tables for Fishers' exact test and Boschloo's test were constructed using the transcription factor regulation target set and significant differentially expressed genes (DEGs) from the log‐phase and PDS‐phase (FDR < 0.05, Wald test, Benjamini‐Hochberg correction). FGSEA was performed using the piano package, version 2.16.0 (Väremo et al. 2013). The cut‐off for significant enrichment for all three methods was set at *p*‐value < 5 × 10^−4^.

## 3 Results

We found that genes pertaining to mitochondrial translation were consistently and significantly upregulated in the *TDA1* deletion mutants. Additionally, Fast Gene Set Analysis (FGSEA) showed that the gene ontology “mitochondrial translation” was enriched to a higher degree during the diauxic shift in the *TDA1* deletion mutant, compared to the reference strain. Finally, transcription factor analysis showed that subunits of the HAP complex, which is a glucose repressed transcription factor responsible for inducing mitochondrial translation, were significantly enriched in the *TDA1* deletion mutant in the log‐phase. Based on the results in this study, we suggest a potential biological role of *TDA1* during the diauxic shift, which is the regulation of mitochondrial translation by repression of HAP complex subunits.

### 3.1 Phenotypical Results

To ensure that the RNAseq samples corresponded to the expected phenotype, OD600, glucose concentration and ethanol concentration was measured when sampling. Most importantly, glucose was expected to be present in the log‐phase and be absent in the PDS‐phase. There were some variations between the experiments, but the condition regarding glucose availability was fulfilled in all experiments, see Table 1.

**Table 1:** Phenotypical data of the sampling point from each experiment.

Time (h)	Strain	Experiment	OD600	Glucose (g/L)	Ethanol (g/L)
0	Reference	1	0.05 ± 0.00		
		2	0.05 ± 0.00		
		3	0.03 ± 0.00		
	*tda1∆*	1	0.05 ± 0.00		
		2	0.04 ± 0.00		
		3	0.04 ± 0.00		
9	Reference	1	1.22 ± 0.00	11.71 ± 0.245	2.86 ± 0.017
		2	0.93 ± 0.05	12.76 ± 1.029	2.50 ± 0.045
		3	0.68 ± 0.07	15.05 ± 0.685	1.35 ± 0.076
	*tda1∆*	1	1.31 ± 0.00	11.81 ± 0.118	2.91 ± 0.109
		2	0.93 ± 0.11	13.96 ± 0.547	2.62 ± 0.113
		3	0.89 ± 0.06	11.94 ± 0.529	2.31 ± 0.202
26	Reference	1	2.46 ± 0.03	N/A	7.27 ± 0.047
		2	2.91 ± 0.08	0.08 ± 0.010	8.81 ± 0.652
		3	3.01 ± 0.02	0.14 ± 0.008	5.98 ± 0.179
	*tda1∆*	1	2.46 ± 0.12	N/A	7.48 ± 0.041
		2	2.79 ± 0.05	0.10 ± 0.012	8.54 ± 0.674
		3	2.96 ± 0.09	0.10 ± 0.010	5.91 ± 0.053

*Note:* The values shown in the “OD600”, “Glucose (g/L)”, and “Ethanol (g/L)” columns are the median ± median absolute deviation of the measured values. Time = 0 refers to sample taken at inoculation, time = 9 is the log‐phase sample, and time = 26 is the PDS‐phase sample. HPLC samples were not taken at time = 0. N/A values indicate that no peak was detected in the software.

Furthermore, to verify that the yeast strains had started consumption of ethanol at 26 h postinoculation, a growth curve experiment replicating the RNAseq experiments was performed, see Figure 1. We found that the ethanol concentration peaked at around 20 h postinoculation, to then slowly decrease over several days. Thus, providing evidence that the yeast cells sampled at 26 h postinoculation were indeed in the PDS‐phase.

### 3.2 Gene Expression Analyses

**Figure 2:**
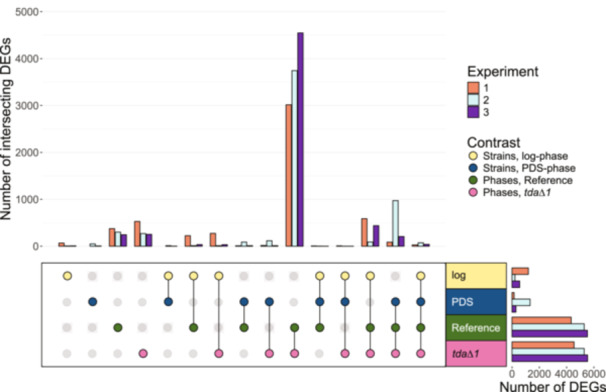
Intersection analysis between contrasts and experiments. UpSet plot of DEGs in the intersections between the four contrasts for each experiment. The horizontal bars represent the total number of DEGs in the contrast while the vertical represent the number of DEGs in the specific contrast intersection, as shown in the matrix beneath the vertical bars.

The gene expression profiles of the three experiments were compared to obtain an overview of the regulatory effect diauxic shift and/or deletion of *TDA1* had on *S. cerevisiae*, see Figure 2. The number of differentially expressed genes between the *tda1∆* mutant and the reference strain during the log‐phase was 1197, 216, and 562 for the first, second, and third experiment, respectively, see Supplementary Table 2.2. The number of differentially expressed genes between the strains during the PDS‐phase was 155, 1325, and 286 for the first, second, and third experiment, respectively, see Supplementary Table 2.3. The number of differentially expressed genes (DEGs) between the phases for the reference strain was 4355, 5230, and 5530 for the first, second, and third experiment respectively, see Supplementary Table 2.4. The number of DEGs between the phases for the *tda1∆* strain was 4545, 5286, and 5538 for the first, second, and third experiment, respectively, see Supplementary Table 2.5. The number of DEGs was greater between the phases for both strains compared to the reference versus *tda1∆* contrasts during either phase. This indicated that the diauxic shift caused a greater impact on the transcriptome compared to the *TDA1* deletion, which was expected.

### 3.3 Transcriptomic Response Over the Diauxic Shift Caused by *TDA1* Deletion

**Figure 3:**
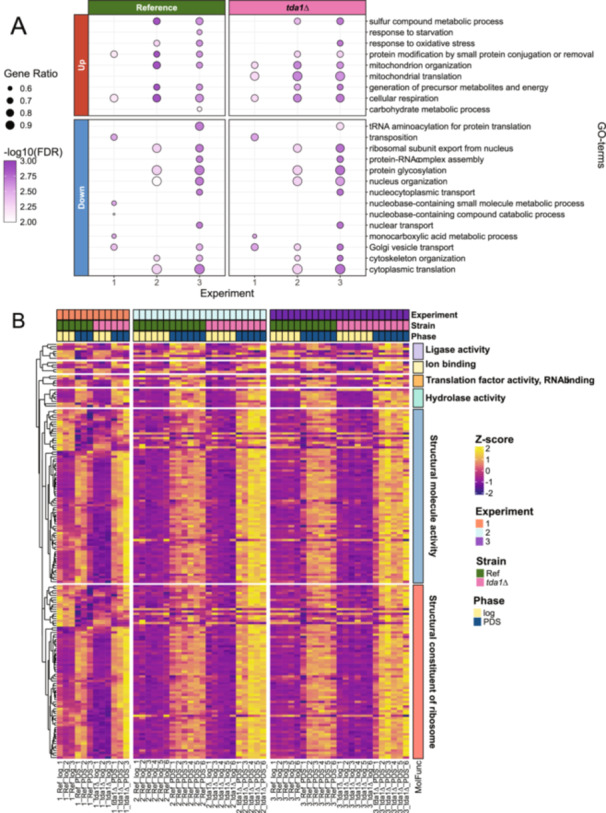
Geneset enrichment analysis. (A) Dot plot of genome set enrichment analysis (FGSEA) on the log‐phase vs PDS‐phase contrasts for the *tda1∆* strain and the reference strain. The y‐axis are the different experiments, x‐axis are GO‐terms that are enriched in at least one contrast, the facet rows separates distinct up‐ or downregulation, the facet columns separates the strains, the dot size is the ratio of DEGs found in the contrast over the total number of genes in the GO term, and the dot colour is set by the adjusted *p*‐value. Gene sets with an adjusted *p*‐value > 0.01 were filtered out. (B) Heatmap based on median of ratios normalised transcript counts within the biological process GO‐term “mitochondrial translation”. The heatmap is separated into experiments (columns) and the molecular function ontologies within the biological process GO‐term “mitochondrial translation” (rows). Genes with multiple molecular functions are shown repeatedly. Hierarchical clustering of genes was performed on the first experiment using Complete‐linkage clustering with Euclidian distance. The genes of the second and third experiments were then ordered based on the hierarchical clustering of the first experiment to highlight differences in expression pattern.

To investigate the role *TDA1* has during the diauxic shift, the gene expression profiles of the reference strain and the *tda1∆* strain over the diauxic shift were examined (log‐phase *vs.* PDS‐phase for each strain). The enriched terms were similar between experiment two and three, but the first experiment stood out as many terms could not be observed with a cutoff of FDR < 0.01, see Figure 3A. The enrichment pattern appeared to be more similar within experiments compared to the *TDA1* deletion. Interestingly however, the enrichment of the gene ontology “mitochondrial translation” was considered statistically significant in the *tda1∆* strain, but not in the reference strain, for all three experiments, see Figure 3A. Among the genes within the biological process GO‐term “mitochondrial translation”, the molecular function GO‐terms “structural molecular activity” and “structural constituent of ribosome” saw a greater trend of upregulation in the *tda1∆* strain compared to the other molecular functions, see Figure 3B. Thus, based on the observation that “mitochondrial translation” was only enriched in *tda1∆* across all three experiments, we hypothesised that Tda1p regulated mitochondrial translation during the diauxic shift. Since the observation from the gene set enrichment analysis was not enough to conclusively prove this hypothesis on its own, other analyses were performed to investigate whether the evidence was consistent with the hypothesis.

**Figure 4:**
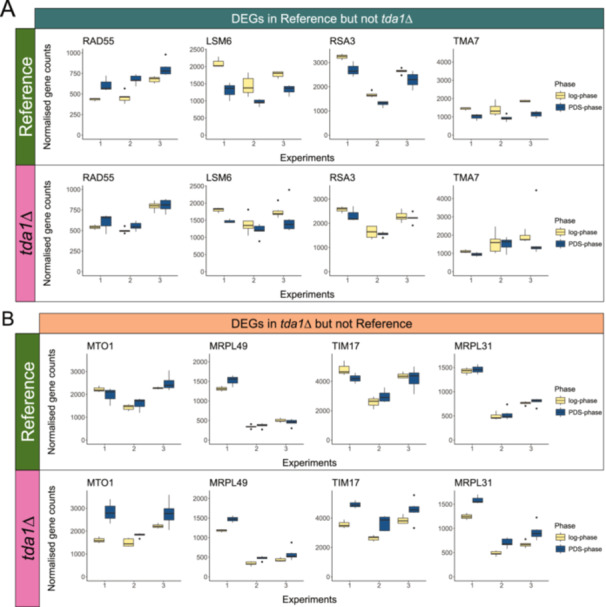
Distribution of genes that were differentially expressed over the diauxic shift. The x‐axis separates the three experiments, the y‐axis is the normalised gene counts, and the colour of the box plots signifies the phase (yellow are log‐phase and blue are PDS‐phase). The centre lines represent the median; box limits represent upper and lower quartiles; whiskers extend to the data points that are furthest from the box limits while within 1.5x interquartile range; dots are data points outside 1.5x interquartile range beyond the box limits. The upper and bottom rows are the gene expressions in the reference strain (green) and *tda1∆* (magenta), respectively. (A) Genes that were consistently differentially expressed in the reference strain but not in *tda1∆*. (B) Genes that were consistently differentially expressed in the *tda1∆* strain but not in the reference.

The intersection of DEGs that were found during the diauxic shift in the reference strain but not in *tda1∆*, and vice versa, were also investigated, see Figure 2. There were 617, 407, and 294 DEGs over the diauxic shift for the reference strain but not for *tda1∆* in experiment one, two, and three, respectively. Out of those DEGs, nine were differentially expressed in all experiments but only 4 were regulated in the same direction in every experiment, see Figure 4A and Supplementary Table 3.1. Notably, *RSA3*, a protein with a likely role in ribosomal maturation, and *TMA7*, a protein of unknown function that associates with the ribosomes, were both related to ribosomes and were both significantly downregulated in the PDS‐phase in the reference strain but not in *tda1∆* (de la Cruz et al. 2004; Fleischer et al. 2006), see Figure 4A. *LSM6*, a subunit of the Lsm1‐Pat1 complex which is involved in cytoplasmic mRNA decay in the nucleolus, was also downregulated, see Figure 4A. No other component in the Lsm1‐Pat1 complex were consistently differentially expressed in only the reference strain. Finally, *RAD55* was upregulated in the reference strain. *RAD55* is a subunit of the heterodimeric Rad55‐Rad57 heterodimer complex that is involved in the recombinational repair of double‐stranded DNA breaks during vegetative growth and also plays a role in telomerase maintenance in the absence of telomerase RNA (Bashkirov et al. 2000; Fortin 2002). The other subunit, *RAD57*, was differentially expressed in both the reference strain and *tda1∆*, suggesting that *TDA1* deletion had no influence on its gene expression. It is interesting to note that all four DEGs that were found in only the reference strain over the diauxic shift were involved in translation or DNA repair and were found in either the nucleus or cytoplasm.

There were 827, 403, and 302 DEGs during the diauxic shift for *tda1∆* but not the reference strain in experiment one, two, and three, respectively, see Figure 2. Out of those DEGs, nine were differentially expressed in all experiments, and all of them were regulated in the same direction in every experiment, see Supplementary Table 3.2. *MRPL49* and *MRPL31*, both mitochondrial ribosomal proteins of the large subunit (Amunts et al. 2014), were upregulated in the PDS‐phase in the *tda1∆* strain but not in the reference, see Figure 4B. *MTO1*, a protein that forms a heterodimer complex with Mss1p to perform 5‐carboxymethyl‐aminomethyl modification to mitochondrial tRNAs (Colby et al. 1998), was upregulated in the deletion mutant but not the reference strain. *MSS1* expression, on the other hand, was not affected by *TDA1* deletion. Upregulation of *MRPL49*, *MRPL31*, and *MTO1* (ribosomal subunits and tRNA modification) were consistent with the theory that the biological process of *TDA1* is to regulate mitochondrial translation. Furthermore, *TIM17*, a subunit in the *TIM27* mitochondrial import translocase complex (Martinez‐Caballero et al. 2007), was also only upregulated in *tda1∆*, see Figure 4B. Thus, four out of the seven DEGs that were only found in the deletion strain over the diauxic shift were located in the mitochondria. The three non‐mitochondrial genes were: *EXO84*, a subunit in the exocytosis and spliceosome (Guo et al. 1999); *GPI19*, subunit of the GPI‐GlcNAc transferase complex located in the endoplasmic reticulum (Newman et al. 2005); and *DCI1*, a protein located in the cytoplasm and peroxisomal matrix whose role as a dodecanoyl‐CoA delta‐isomerase in fatty acid metabolism is disputed (Ntamack et al. 2009), see Supplementary Table 3.2. Note that no paralogs or other subunits in their respective complexes were consistently differentially expressed in *tda1∆* but not reference over the diauxic shift.

### 3.4 The Effect of *TDA1* Deletion During log‐Phase and PDS‐Phase

Differential gene expression was investigated between the *tda1∆* mutant and the reference strain during each phase separately. The number of DEGs during the log‐phase in each experiment ranged between 216 and 1197, but only six genes were differentially expressed in all three experiments, see Table 2. Out of those six genes, only four genes had the same direction of regulation between the experiments, see Table 2. Interestingly, *HXT6* was upregulated in the log‐phase in all three experiments. *HXT6* is high‐affinity glucose transporter which is repressed through the Snf3p/Rgt2p signalling pathway during high glucose concentrations (Kayikci and Nielsen 2015). The paralog *HXT1*, *HXT7* (whose ORF is nearly identical to *HXT6*), and *HXT4* (paralog to HXT7) were differentially expressed in some experiments but not all (Byrne and Wolfe 2005; Diderich et al. 1999; Reifenberger et al. 1995), see Supplementary Table 2.1. The upregulation of *COS12*, an member of a group of endosomal proteins involved in the degradation of plasma membrane proteins (MacDonald et al. 2015). Out of the 11 genes in the COS family, only *COS12* was consistently differentially expressed across the experiments. The downregulated genes during the log‐phase were *SNZ1* and *YPR204W*. *SNZ1* is involved in pyridoxine (vitamin B6) synthesis (Rodríguez‐Navarro et al. 2002). Interestingly, none of its paralogs *SNZ2* or *SNZ3* were differentially expressed in any experiment. Furthermore, *SNZ1* is usually induced during the post diauxic shift phase (Braun et al. 1996). *YPR204W* is a DNA helicase located in the telomeric Y' element and is induced when telomerase is defective (Yamada et al. 1998).

**Table 2:** Differentially expressed genes (FDR < 0.05) between the t*da1∆* mutant and reference strain during log‐phase and PDS‐phase that were consistently differentially expressed in all three experiments.

Phase	Systematic name	Standard name	Origin of name	Direction	Mean ± standard deviation of log2 fold change
log‐phase	YDR343C	HXT6	HeXose transporter	UP	1.27 ± 0.92
	YGL263W	COS12	Conserved sequence	UP	1.50 ± 0.73
	YMR096W	SNZ1	SnooZe	DOWN	−0.42 ± 0.14
	YPR204W	—	—	DOWN	−0.43 ± 0.11
	YDL027C	MRX9	Mitochondrial oRganization of gene eXpression	MIXED	—
	YHL036W	MUP3	Methionine uptake	MIXED	—
PDS‐phase	YBL021C	HAP3	Heme activator protein	UP	0.29 ± 0.12
	YBL059W	IAI11	Interactor of Aim11	UP	0.22 ± 0.05
	YGL063W	PUS2	PseudoUridine synthase	UP	0.32 ± 0.19
	YML087C	AIM33	Altered inheritance of mitochondria	UP	0.42 ± 0.10
	YLR032W	RAD5	RADiation sensitive	DOWN	−0.21 ± 0.05
	YER045C	ACA1	ATF/CREB activator	DOWN	−0.29 ± 0.10

Differential gene expression was also investigated between the *tda1∆* and reference strain during the PDS phase for each experiment. The number of DEGs during the PDS‐phase in each experiment ranged between 155 and 1325, but only six genes were differentially expressed in all three experiments, see Figure 2 and Table 2. All six genes had the same direction of regulation between each experiment, see Table 2. *RAD5* is a DNA helicase/Ubiquitin ligase involved in DNA repair and associates with telomeres in absence of telomerase (Fallet et al. 2014). Interestingly, three genes that are thought to be related to mitochondrial function, but not very well understood, were all upregulated in all three experiments, namely *AIM33*, *IAI11*, and *PUS2*. *AIM33* is a protein with an unknown function which is highly conserved across species (Hess et al. 2009). It has been observed that *AIM33* deletants display reduced frequency of spontaneous mitochondrial genome loss and is also unable to grow on medium containing non‐fermentable carbon sources (Hess et al. 2009, p. 200). *IAI11* is also a protein with unknown function that has been detected to a higher extent in highly purified mitochondria and it would thus be reasonable to assume that its function is related to the mitochondria (Morgenstern et al. 2017). *PUS2* is a mitochondrial pseudouridine synthase that modifies mitochondrial mRNA and *pus2∆* have impaired growth rate but improved biomass yield when grown on synthetic minimal media, which could be due to the deletants using the carbon source more efficiently or a loss of the ability to induce cell cycle arrest under growth conditions the wildtype strain has considered unfavourable.

Also worth noting is that two out of the six consistently significantly differentially expressed genes during the PDS‐phase were transcription factors, *ACA1* and *HAP3*. *ACA1* was downregulated and is a ATF/CREB family basic leucine zipper (bZIP) transcription factor and is thought to be important for carbon utilisation (Garcia‐Gimeno and Struhl 2000). *HAP3* was upregulated and is subunit of the Hap complex which is a heme‐activated and glucose repressed transcription factor that regulates expression of respiratory genes and mitochondrial translation. The upregulation of *HAP3* is especially interesting as it is consistent with the evidence presented in previous sections, namely the enrichment of genes in the GO‐term “mitochondrial translation” and upregulation of genes related to mitochondrial translation (*MRPL31, MRPL49*, and *MTO1*). The increased transcript counts of *HAP3*, and by extension the activity of the HAP‐complex, could be an explanation to the increased expression of genes related to mitochondrial translation in the *TDA1* deletion mutant. Therefore, if the HAP‐complex regulated genes are enriched and upregulated in the *tda1∆* strain, it would suggest that *TDA1* regulates mitochondrial translation by repressing the activity of the HAP‐complex.

### 3.5 Transcription Factor Analysis

To assess the interaction between *TDA1* and the HAP‐complex, the expression of Hap4p‐regulated genes was analysed. Hap4p is the DNA binding component and the principal activation function of the complex (Forsburg and Guarente 1989). It was thus hypothesised that differential expression of HAP3, which is crucial to the function of the complex but not the main DNA binding component, would ultimately affect the expression of Hap4p‐regulated genes. Furthermore, to assess the previous Mig1p‐mediated glucose repression model, the expression of Mig1p repressed genes was also investigated. The Mig1p‐repressed genes were statistically enriched in experiment one during the log‐phase using FGSEA while not being statistically enriched in any other contrast, see Table 3. Conversley, Hap4p regulated genes were statistically enriched during the PDS‐phase in all three experiments using FGSEA, see Table 4. However, the enrichment of Hap4p regulated genes were not statistically enriched in experiment 3 during the PDS‐phase using Fisher's exact test or Boschloo's test. The evidence from this study show that it is more likely that Tda1p (and its phosphorylation target Hxk2p) is involved with the HAP‐complex repression rather than Mig1p‐mediated glucose repression.

**Table 3:** Statistical analyses of enrichment of Mig1p‐regulated genes.

Method	Experiment	log‐phase (*p*‐value)	PDS‐phase (*p*‐value)
Fisher	1	9.67E‐01	6.45E‐01
	2	4.19E‐01	5.20E‐01
	3	3.08E‐03	8.55E‐01
Boschloo	1	1.63E‐01	1.00E + 00
	2	6.48E‐01	8.45E‐01
	3	2.82E‐03	1.00E + 00
FGSEA	1	9.91E‐01	*3.97E‐04
	2	3.76E‐01	1.43E‐01
	3	3.50E‐02	7.64E‐01

*Note:* The *p*‐value from the FGSEA method are FDR‐corrected and only distinct upregulation was considered. The *p*‐value cut‐off for enrichment to be considered statistically significant was set to *p*‐value < 5 × 10^−4^. Enrichments that are considered statistically significant under these conditions are marked with an asterisk (*).

**Table 4:** Statistical analyses of enrichment of Hap4p‐regulated genes.

Method	Experiment	log‐phase (*p*‐value)	PDS‐phase (*p*‐value)
Fisher	1	9.07E‐01	*5.93E‐08
	2	5.56E‐01	*8.90E‐07
	3	7.40E‐03	4.46E‐01
Boschloo	1	3.03E‐01	*4.34E‐08
	2	7.01E‐01	*7.60E‐07
	3	6.82E‐03	7.27E‐01
FGSEA	1	2.38E‐02	*3.97E‐04
	2	8.22E‐02	*3.50E‐04
	3	5.71E‐04	*4.25E‐04

*Note:* The *p*‐values from the FGSEA method are FDR‐corrected and only distinct upregulation was considered. The p‐value cut‐off for enrichment to be considered statistically significant was set to *p*‐value < 5 × 10^−4^. Enrichments that are considered statistically significant under these conditions are marked with an asterisk (*).

## 4 Discussion

Metabolic network adaptations caused by changing nutrient availability or stress responses are complex. Despite decades of research (De Deken 1966), the regulatory network enabling the diauxic shift in *S. cerevisiae* is still not completely mapped out (Brunnsåker et al. 2023). While the basic regulatory mechanism during diauxic shift is quite well understood, the subtle yet relevant effects caused by minor regulatory proteins remains poorly understood. In this study we performed transcriptomic analysis to investigate the role of *TDA1* during the diauxic shift. Due to the sensitivity of transcriptomics and biological systems (especially transient phenomena like the diauxic shift, we expected uncertainty in the RNAseq data. Previous studies have also discussed the difficulty of reproducing results from bulk RNAseq experiments (Li et al. 2022; McIntyre et al. 2011; SEQC/MAQC‐III Consortium 2014). We therefore performed the same experiment three times to assess the reproducibility of the experiments and increase the robustness of our conclusions. We found that there was indeed noticeable variation in the RNAseq data between the experiments. The cause of this variation could be due to slightly different initial cultivation conditions, difference between RNA extraction kit batches, library preparation, sequencing instruments, etc. While some variations could be seen in the phenotypical data, we have no reason to believe that the samples did not belong to either the log‐phase or PDS‐phase as the observed glucose levels corresponded with the expected outcome, see Figure 1 and Table 1. Instead, we argue that observations which are consistent across different sequencing facilities leads to conclusions that are more replicable and robust.

**Figure 5:**
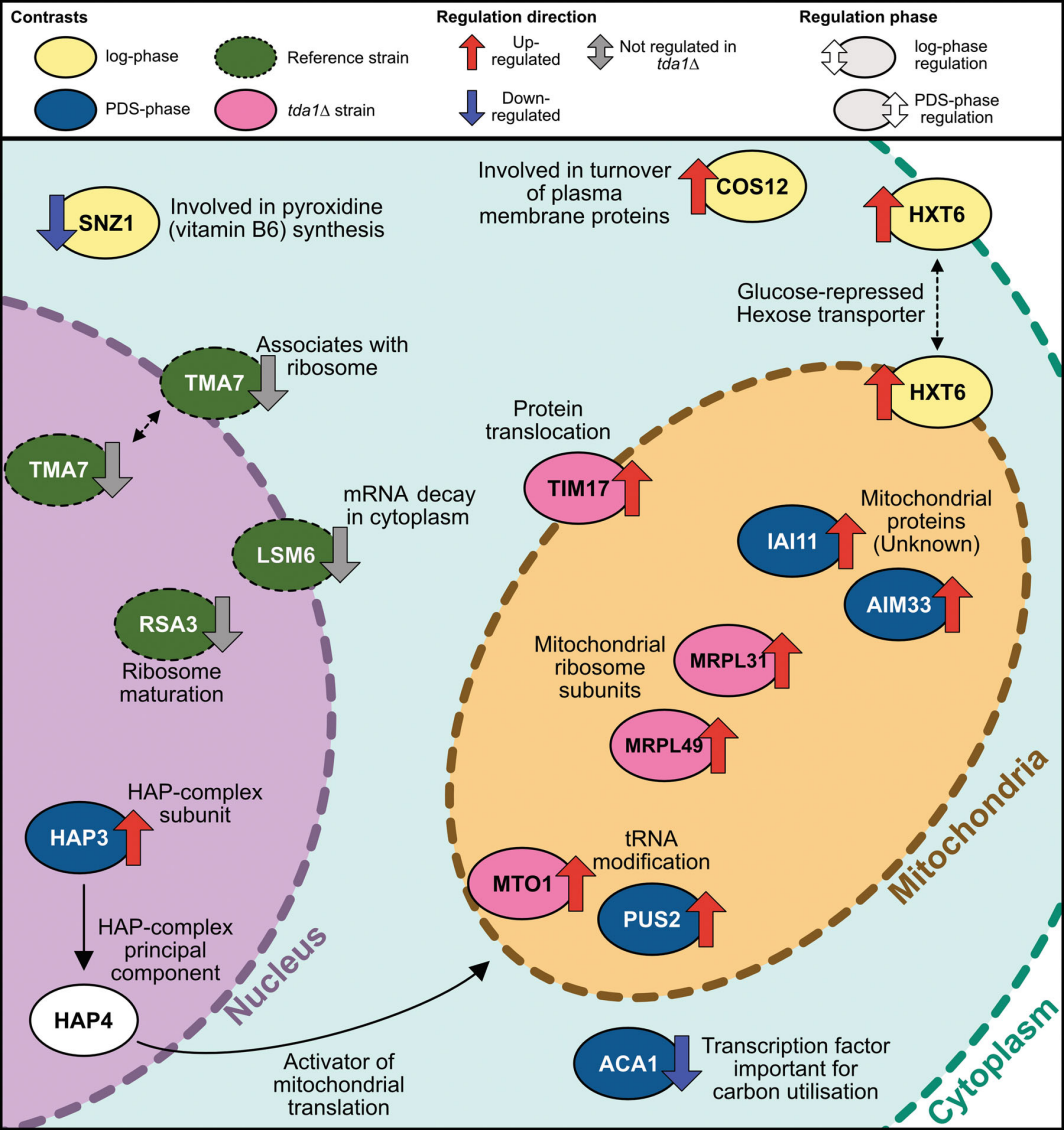
Graphical summary of the key findings in this study. The oval shapes represent significantly differentiated genes; colour represent in which contrast the genes were differentially expressed (yellow = Strains, log‐phase; blue = strains, PDS‐phase, green = Phases, reference but not *tda1∆*, and magenta = Phases, *tda1∆* but not reference). The arrows represent regulation (red upwards arrow = upregulated in *tda1∆*; blue downwards arrow = downregulated in *tda1∆*; grey arrows = regulation that occurred in the reference strain but not *tda1∆* during the diauxic shift). The location of the arrow represents the phase in which the gene was differentially expressed (left = log‐phase; right = PDS‐phase).

Combining findings from previous studies with the evidence presented in this study, we propose a hypothesis that states that Hxk2p phosphorylation by Tda1p regulates mitochondrial translation by repressing the activity of the HAP‐complex. Previous studies have shown that Tda1p is essential for Hxk2p phosphorylation (Müller et al. 2022) and phosphorylated Hxk2p is introduced into the nucleus during low abundance of glucose (Lesko et al. 2023). We would therefore expect an absence of Hxk2p in the nucleus after the diauxic shift in a *tda1∆* mutant. If phosphorylated Hxk2p then acts as a transcription coregulator, it would follow that its transcription targets would be differentially expressed in the *tda1∆* mutant. The gene ontology enrichment analysis using biological process gene sets showed that mitochondrial translation was consistently enriched in the *tda1∆* mutant during the PDS‐phase, but not in the reference; suggesting that the mitochondrial translation was repressed in the presence of *TDA1*. Furthermore, we found that genes that participate in mitochondrial translation such as *MTO1, PUS2, MRPL31*, and *MRPL49* were upregulated in the *tda1∆* mutant during the PDS‐phase, which was consistent with the proposed hypothesis, see Figure 5.

The products of the genes *IAI11* and *AIM33* are also located in the mitochondria, and the upregulation of these genes could be the result of the increased mitochondrial translation activity in the *tda1∆* strain. Furthermore, *HAP3* was consistently upregulated in the *tda1∆* mutant during the PDS‐phase. This is interesting as it is a subunit of the HAP‐complex, which in turn regulate mitochondrial translation during the diauxic shift. Enrichment analysis of the principal component in the HAP‐complex, *HAP4*, revealed that *HAP4* regulated genes were enriched in the PDS‐phase, providing further evidence for the role of *TDA1* in regulating mitochondrial translation and a potential mechanistic pathway for the suggested regulation. In our previous metabolomics study on *TDA1* we found that the activity in the sphingolipid biosynthesis pathway was significantly enriched during the PDS‐phase (Brunnsåker et al. 2023). Sphingolipids are known to regulate mitochondrial function (Spincemaille et al. 2014) and an enrichment of metabolites in the sphingolipid biosynthesis pathway would be expected if the mitochondrial activity was affected by the *TDA1* deletion. The results from this transcriptomics study and our previous metabolomics study are thus consistent.

The role of *TDA1* during the log‐phase was less clear. *HXT6* is glucose repressed hexose transporter whose expression is induced during the diauxic shift (Kayikci and Nielsen 2015). Thus, it would seem reasonable to assume that the diauxic shift occurred earlier in the *tda1∆* mutant since *HXT6* was upregulated in the log‐phase. However, *SNZ1*, an enzyme which is induced during the diauxic shift (Braun et al. 1996), was downregulated. Since two genes whose induction acts a marker for the diauxic shift were consistently regulated in different directions, it is therefore unlikely that the absence of *TDA1* caused the diauxic shift to happen earlier in the deletion mutant. Instead, it is more likely that the differential expression of these two genes in the log‐phase is caused by an unknown mechanism.

Genes related to telomere abnormality were downregulated in the *tda1∆* mutant compared to the reference strain. *RAD5* and *YPR204W* were downregulated in the *tda1∆* mutant during the log‐phase and *RAD55* was only upregulated over the diauxic shift in the reference strain, see Table 2 and Figure 3A. Telomeres are the nonprotein coding repeated DNA sequences found at the ends of the chromosomes and is most found in eukaryotes (Wellinger and Zakian 2012). Their function is to protect the terminal regions of the chromosomes from progressive degradation and have thus been used as a biomarker for aging (Vaiserman and Krasnienkov 2021). Heightened telomerase and abnormal extension of the telomeres is characteristic of cancer cells in higher eukaryotes (Jafri et al. 2016). Whether the heightened expression of genes related to telomere abnormality was caused by a defect in our BY4741 strain or by the absence of *TDA1* during the diauxic shift is unknown. It is worth noting however that previous studies have found that the expression of telomeric repeat‐containing RNA increases during the diauxic shift (Perez‐Romero et al. 2018). Furthermore, the sphingolipid biosynthesis pathway enrichment found in our previous metabolomic study (Brunnsåker et al. 2023) is consistent with altered telomere activity observed in our study as sphingolipid metabolism is linked to cell aging and telomere clustering (Ikeda et al. 2015; Spincemaille et al. 2014).

It is unlikely that *TDA1* participates in Mig1p‐mediated glucose repression. Three types of statistical tests were used for the transcription factor analysis: Fisher's exact test, Boschloo's test, and FGSEA. The ambiguous enrichment of Mig1p repressed genes in experiment one and Hap4p in experiment three followed a similar pattern where it was considered statistically significant using FGSEA while not being statistically significant using the contingency table‐based tests. However, our confidence in the theory that *TDA1* is linked with the expression HAP‐complex is strengthened by the fact that the results from the two other experiments suggested that Hap4p enrichment was statistically significant. Conversely, our confidence in the Mig1p model was weakened by the fact that its enrichment was not considered to be statistically significant in any of the other experiments using any of the tests. This demonstrates the necessity of repeating experiments when the observed expressions are expected to be noisy and subtle.

The cultivation media was not buffered and there is a risk that there was a significant pH decrease as the biomass in the culture increased and organic acids leaked out (Sigler and Höfer 1991). However, the transcriptional changes in this study does not resemble the expected transcription profile observed in low extracellular pH environments (Dong et al. 2017). Therefore, the risk that a potential acidification of the medium served as a confounding factor should be minimal. Furthermore, it is well known that the S288c strain and its derivatives (e.g. BY4741) have an abnormal mitochondrial biology due to a mutated copy of *HAP1* (Gaisne et al. 1999). While we are confident that *TDA1* deletion in the BY4741 background leads to overexpression of subunits in the HAP‐complex and increased mitochondrial translation, it is uncertain whether the conclusions drawn in this study can be generalised to other yeast strains and eukaryotes in general. To extrapolate our conclusion to other yeast strains, it would require study on a non‐S288c derived *S. cerevisiae* strain. Finally, the *TDA1* deletion strain used in this study (Y00878) has been reported to possess an abnormal repetitive *CUP1‐*locus copy number (Puddu et al. 2019). While this suggests that some kind of secondary compensatory mutation has occurred in the strain, it is worth noting that the main role of *CUP1* is maintaining copper homeostasis, which is unlikely to affect the conclusions drawn in this study. In fact, Puddu et al. (2019) reports that the mitochondrial DNA copy‐numbers were unchanged in the *tda1∆*/Y00878 strain. Therefore, it seems unlikely that our conclusions on the relation between *TDA1* and mitochondrial translation were caused by secondary compensatory mutations.

Based on the RNAseq data presented in this study, we propose a model in which Tda1p and its phosphorylation target Hx2p regulates mitochondrial translation through repression of the HAP‐complex during the diauxic shift. The purpose of this regulation is unknown, as it would seem counter‐intuitive to repress a metabolic rewiring that is required for *S. cerevisiae* to survive in glucose depleted environments. An explanation could be that nuclear localisation of Hxk2p acts a hedge against potential reintroduction of environmental glucose. Rewiring the metabolism from anaerobic fermentation to aerobic respiration is an expensive energy investment for yeast (Di Bartolomeo et al. 2020). In addition, the nutrient availability in nature tends to fluctuate and it is not uncommon that glucose is reintroduced into the environment by fungi that breaks down starch into monosaccharides. Thus, it follows that cells that invest into respiration on ethanol too quickly would be outcompeted by cells that remained in anaerobic fermentation if glucose is suddenly reintroduced. Additionally, the cells would also save energy that would be spent producing new Hxk2p enzymes, if Hxk2p is dephosphorylated and returns to participating in the glycolysis when glucose is reintroduced. Investigating this hypothesis is outside the scope of this study, but for future work we suggest lag‐time analyses where *tda1∆* and a reference strain in the PDS‐phase are inoculated into glucose rich media. Additionally, we suggest measuring Hxk2p abundance in cells that are reintroduced to glucose rich environments to assess the theory that Hxk2p is localised into the nucleus to save translation costs of key glycolytic enzymes.

Our suggested hypothesis relies on some unverified assumptions. First, our hypothesis describes a protein interaction pathway that was inferred using transcriptomic data, i.e. indirect evidence. While previous studies have verified Hxk2p phosphorylation by Tda1p and nuclear introduction of phosphorylated Hxk2p during glucose deprived conditions (Lesko et al. 2023; Müller et al. 2022), the link between nuclear phosphorylated Hxk2p and *HAP3* expression remains unverified. Our assumption is that phosphorylated Hxk2p acts as a coregulator to a transcription factor. To verify this assumption, it would require protein‐protein interaction studies using phosphorylated monomeric Hxkp2, which is outside the scope of this study. We therefore refer this to future work, as this data has yet to be generated. Second, our study observed the transcriptomic consequences of *TDA1* deficiency during the diauxic shift. We made a prior assumption based on previous studies that the prominent role of TDA1 is during diauxic growth (Brunnsåker et al. 2023; Kaps et al. 2015). However, it is also possible that there are other environmental conditions wherein Tda1p is more prominent. For example, Müller et al. suggests that *TDA1* could be involved in the oxidative stress response, which is also induced as *S. cerevisiae* switches to respiratory metabolism post diauxic shift. We therefore also suggest future studies of *TDA1* under different environmental conditions to explore whether the naturally selected function of *TDA1* lies elsewhere.

## 5 Conclusion

To conclude, the evidence from this study is consistent with the recent studies that argue that Tda1p and its phosphorylation product Hxk2p does not associate with Mig1p (Lesko et al. 2023; Müller et al. 2022). We found that genes related to mitochondrial translation were significantly enriched and upregulated in the PDS‐phase for the *TDA1* deletion mutant. This observation is consistent with the enriched activity of sphingolipid biosynthesis pathway we observed in our previous metabolomics study. We also found that *HAP3* was consistently upregulated in the deletion mutant during the PDS‐phase. Furthermore, genes regulated by the principal component of the HAP‐complex, *HAP4*, were enriched in the *TDA1* deletion mutant during the PDS‐phase. Based on these observations, we suggest a hypothesis that states that *TDA1* regulates mitochondrial translation during the diauxic shift by repressing the HAP‐complex.

## Author Contributions

Conceived and designed the experiments: Erik Y. Bjurström, Ievgeniia A. Tiukova, Ross D. King Performed experiments: Erik Y. Bjurström, Praphapan Lasin Processed RNAseq sample: E.Y.B, Praphapan Lasin Processed and analysed the data: Erik Y. Bjurström Wrote the paper: Erik Y. Bjurström, Daniel Brunnsåker, Ievgeniia A. Tiukova, Ross D. King. All authors reviewed the manuscript.

## Conflicts of Interest

The authors declare no conflicts of interest.

## Supporting information

Supplementary table 1: Growth data.

Supplementary table 2.1: Raw counts for experiment 1, 2, and 3.

Supplementary table 2.2: Significantly differentially expressed genes, ref v mutant, log‐phases.

Supplementary table 2.3: Significantly differentially expressed genes, ref v mutant, PDS‐phases.

Supplementary table 2.4: Significantly differentially expressed genes, log v PDS, ref.

Supplementary table 2.5: Significantly differentially expressed genes, log v PDS, mutant.

Supplementary table 3.1: Significantly differentially expressed genes, log v PDS, ref but not mutant.

Supplementary table 3.2: Significantly differentially expressed genes, log v PDS, mutant but not ref.

## Data Availability

Data deposition: RNA‐seq data has been submitted in the form of raw reads in the form of. fastq files under the accession number PRJEB59812 at the European Nucleotide Archive (ENA). All code required for reproduction of the analysis and figures in the study can be found on GitHub at https://github.com/erikbju/TDA1_proj. Data deposition: RNA‐seq data has been submitted to the European Nucleotide Archive in the form of raw reads in the form of. fastq files under the accession number PRJEB59812.
